# The CXCL10/CXCR3 Axis Promotes Disease Pathogenesis in Mice upon CVA2 Infection

**DOI:** 10.1128/spectrum.02307-21

**Published:** 2022-05-23

**Authors:** Ruonan Liang, Shuaiyin Chen, Yuefei Jin, Ling Tao, Wangquan Ji, Peiyu Zhu, Dong Li, Yu Zhang, Weiguo Zhang, Guangcai Duan

**Affiliations:** a Department of Epidemiology, College of Public Health, Zhengzhou University, Zhengzhou, China; b School of Public Health, Xinxiang Medical University, Xinxiang, China; c Suzhou Institute of Systems Medicine, Chinese Academy of Medical Sciences, Suzhou, China; d Henan Key Laboratory of Molecular Medicine, Zhengzhou University, Zhengzhou, China; Wuhan Institute of Virology

**Keywords:** coxsackievirus A2, hand-foot-and-mouth disease, CXCL10, CXCR3

## Abstract

Coxsackievirus A2 (CVA2) is an emerging pathogen that results in hand-foot-and-mouth disease (HFMD) outbreaks. Systemic inflammatory response and central nervous system inflammation are the main pathological features of fatal HFMD. However, the immunopathogenesis of CVA2 infection is poorly understood. We first detected the transcriptional levels of 81 inflammation-related genes in neonatal mice with CVA2 infection. Remarkably, CVA2 induced higher expression of chemokine (C-X-C motif) ligand 10 (CXCL10) in multiple organs and tissues. CXCL10 acts through its cognate receptor chemokine (C-X-C motif) receptor 3 (CXCR3) and regulates immune responses. CXCL10/CXCR3 activation contributes to the pathogenesis of many inflammatory diseases. Next, we found CXCL10 and CXCR3 expression to be significantly elevated in the organs and tissues from CVA2-infected mice at 5 days postinfection (dpi) using immunohistochemistry (IHC). To further explore the role of CXCL10/CXCR3 in CVA2 pathogenesis, an anti-CXCR3 neutralizing antibody (αCXCR3) or IgG isotype control antibody was used to treat CVA2-infected mice on the same day as infection and every 24 h until 5 dpi. Our results showed that αCXCR3 therapy relieved the clinical manifestations and pathological damage and improved the survival rate of CVA2-infected mice. Additionally, αCXCR3 treatment reduced viral loads and reversed the proinflammatory cytokine (interleukin 6 [IL-6], tumor necrosis factor alpha [TNF-α], and IL-1β) expression, apoptosis, and inflammatory cell infiltration induced by CVA2. Collectively, our study presents evidence for the involvement of the CXCL10/CXCR3 axis in CVA2 pathogenesis. The activation of CXCL10/CXCR3 contributes to CVA2 pathogenesis by inducing apoptosis, proinflammatory cytokine expression, and inflammatory cell infiltration, which can be reversed by αCXCR3 therapy. This study provides new insight into the pathogenesis of HFMD, which has an important guiding significance for the treatment of HFMD.

**IMPORTANCE** Systemic inflammatory response and central nervous system inflammation are the main pathological features of fatal HFMD cases. We detected the expression of 81 inflammation-related genes and found higher expression of CXCL10 in CVA2-infected mice. Next, we confirmed CXCL10/CXCR3 activation using immunohistochemistry and found that anti-CXCR3 neutralizing antibody (αCXCR3) therapy could relieve the clinical manifestations and pathological damage and improve the survival rate of CVA2-infected mice. Additionally, αCXCR3 treatment reduced viral loads and reversed the proinflammatory cytokine (IL-6, TNF-α, and IL-1β) expression, apoptosis, and inflammatory cell infiltration induced by CVA2. Collectively, our study presents the first evidence for the involvement of the CXCL10/CXCR3 axis in CVA2 pathogenesis. The activation of CXCL10/CXCR3 contributes to CVA2 pathogenesis via inducing apoptosis, proinflammatory cytokine expression, and inflammatory cell infiltration, which can be reversed by αCXCR3 therapy. This study provides new insight into the pathogenesis of HFMD, which has an important guiding significance for the treatment of HFMD.

## INTRODUCTION

Hand-foot-and-mouth disease (HFMD) is a common infectious disease caused by enterovirus (EV) infection and occurs most frequently in children under 5 years old (1). Although enterovirus 71 (EV71) and coxsackievirus A16 (CVA16) are recognized as the main causative pathogens, an increasing number of HFMD patients with coxsackievirus A2 (CVA2) infection have been reported in recent years (2). In 2009 to 2012, there was an infant reported with neurological complications due to CVA2 infection in Taiwan (3). In 2012, CVA2 was found in 2 dead children who had had severe upper respiratory tract infections in Hong Kong (4); however, fatal CVA2 infections are rare relative to fatal EV71 infections. High prevalences of CVA2 also occurred in mainland China (5), South Korea (6), Thailand (7), and Brazil (8). Therefore, CVA2 has become an important public health issue deserving greater attention. Generally, HFMD infections are self-limited, but some infections develop into encephalitis and cardiopulmonary dysfunction. A mass of immune cells and inflammatory cytokines play an important role in the immunopathogenesis of HFMD (9). The activation of immune cells and immune responses provides immune protection against EV infection. However, the overactivation of immune cells and, subsequently, excessive inflammatory cytokine production lead to tissue damage. Previous studies had examined features of the expression of cytokines and chemokines in severe HFMD patients and found that interleukin 1β (IL-1β), IL-6, IL-10, chemokine (C-X-C motif) ligand 10 (CXCL10), and tumor necrosis factor (TNF) in peripheral fluids were significantly increased compared with their levels in mildly ill patients (10, 11). Among them, CXCL10 was considered a new detection biomarker for HFMD, with high sensitivity and specificity (12). CXCL10, a 10-kDa protein, is categorized functionally as a Th1 chemokine. The secretion of CXCL10 by cluster of differentiation 4-expressing (CD4^+^), CD8^+^ natural killer (NK) cells is dependent on gamma interferon (IFN-γ) (13). Under the stimulation of IFN-γ, CXCL10 is also produced by endothelial cells, fibroblasts, keratinocytes, thyrocytes, preadipocytes, etc. (14) CXCL10 initiates its function via interacting with a 7-transmembrane receptor coupled to G proteins, chemokine (C-X-C motif) receptor 3 (CXCR3). CXCL10 and its receptor, CXCR3, appear to contribute to the pathogenesis of many inflammatory diseases (15). More and more attention has been devoted to understanding their activities as proinflammatory mediators (16).

In the present study, we established a neonatal mouse model of CVA2 infection and found higher expression of CXCL10. To further investigate the role of the CXCL10/CXCR3 axis in CVA2 pathogenesis, we verified the expression of CXCL10 and CXCR3 in multiple organs and tissues and used an anti-CXCR3 neutralizing antibody (αCXCR3) to define its therapeutic effect. Our study will be beneficial for understanding the pathogenesis of HFMD and to better inform and plan clinical trials.

## RESULTS

### CVA2 induces higher expression of CXCL10 in a neonatal mouse model.

**FIG 1:**
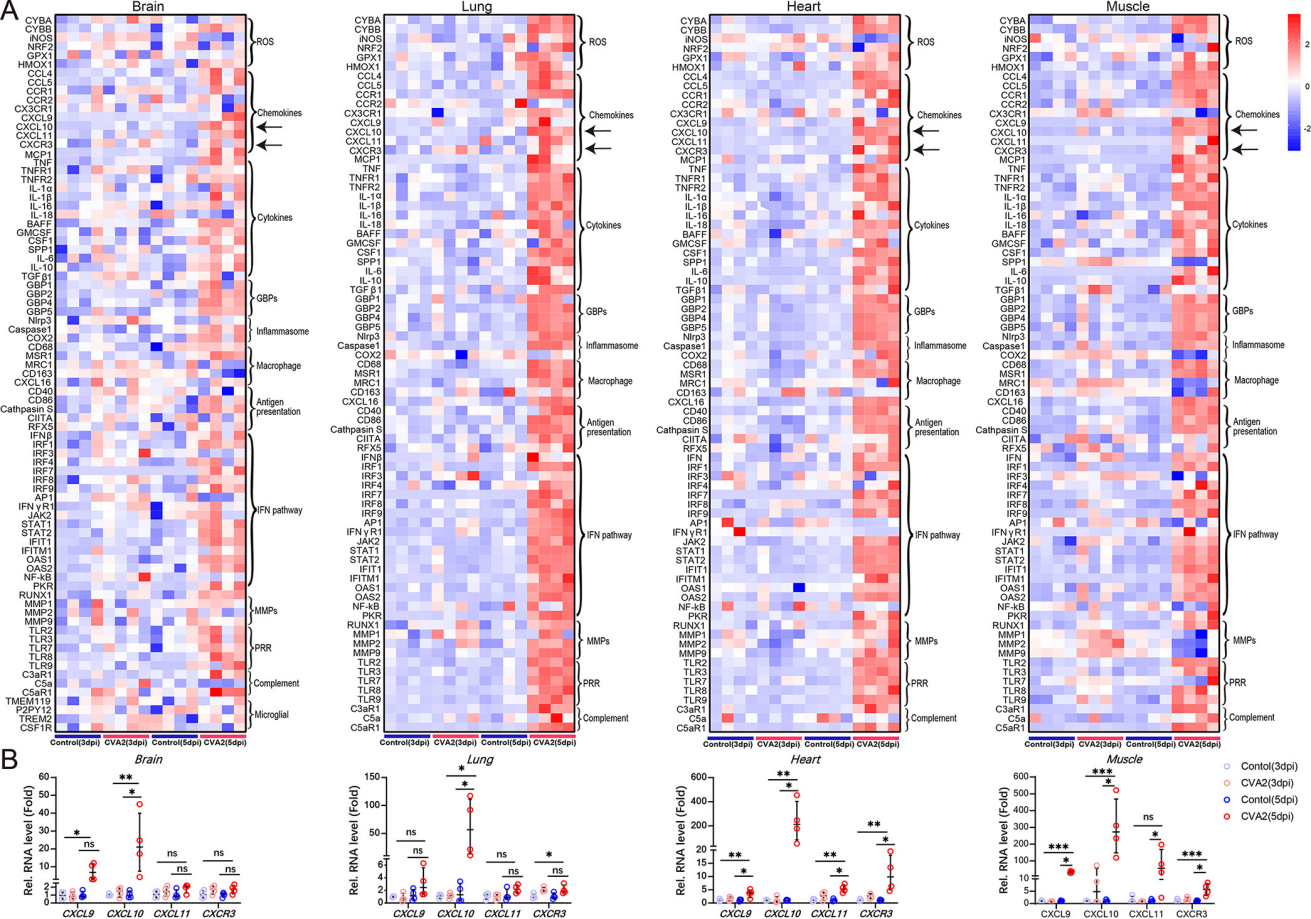
Visualization of inflammation-related genes during CVA2 infection. (A) Expression profiles of inflammation-related genes in organs and tissues (brains, lungs, hearts, and skeletal muscles) of control and CVA2-infected mice. (B) Transcription levels of the most significantly upregulated gene (CXCL10), its structural homologous molecules (CXCL9 and CXCL11), and its receptor (CXCR3) in the organs and tissues. *n* = 4 per group. ns, not significant; *, *P < *0.05; **, *P < *0.01; ***, *P < *0.001.

Uncontrolled inflammation is responsible for the disease severity of HFMD. To investigate the inflammatory response following viral infection, the expression of inflammation-related genes during CVA2 infection was detected by reverse transcription-quantitative PCR (qRT-PCR). As shown by the results in Fig. 1, we screened all inflammation-related genes with roles in oxidative stress, chemokines, inflammatory cytokines, guanylate binding proteins (GBPs), inflammasomes, macrophage, antigen presentation, IFN signaling pathway, matrix metalloproteinases (MMPs), pattern recognition receptors (PRRs), complement, and microglial activation in organs and tissues (brains, lungs, hearts, and skeletal muscles) of control and CVA2-infected mice. The expression of genes encoding cytochrome *b*-245 beta polypeptide (CYBB) (oxidative stress), CD68 (macrophage), proinflammatory cytokines (TNF-α, TNF receptor 2 [TNFR2], IL-6, IL-1β, and IL-18), BAFFs related to B-cell function, inflammasomes (GBPs, NLR family pyrin domain containing 3 [NLRP3], and Caspase-1), MMPs, chemokines (MCP-1 [chemokine {C-C motif} ligand 2], C-C motif chemokine ligand 5 [CCL5], CCL4, and CXCL10), antigen processing and presentation (macrophage scavenger receptor 1 [MSR1], CD86, and cathepsin S), and innate immunity (Toll-like receptor 3 [TLR3], TLR7, TLR8, TLR9, IFN regulatory factor 1 [IRF1], IRF7, IRF8, IRF9, interferon alpha and beta receptor subunit 1 [IFNAR1], signal transducer and activator of transcription 1 [STAT1], STAT2, Janus kinase [JAK], oligoadenylate synthetase 1 [OAS1], OAS2, protein kinase R [PKR], interferon-induced protein with tetratricopeptide repeats 1 [IFIT1], and interferon-induced transmembrane protein 1 [IFITM1]) were significantly elevated compared to their levels in controls. Importantly, we found that the expression levels of CXCL10 in the above-named organs and tissues were all upregulated significantly, by 20 to 300 times. Our results suggest that higher expression of CXCL10 may participate in CVA2 pathogenesis.

### CVA2 infection leads to CXCL10/CXCR3 axis activation.

**FIG 2:**
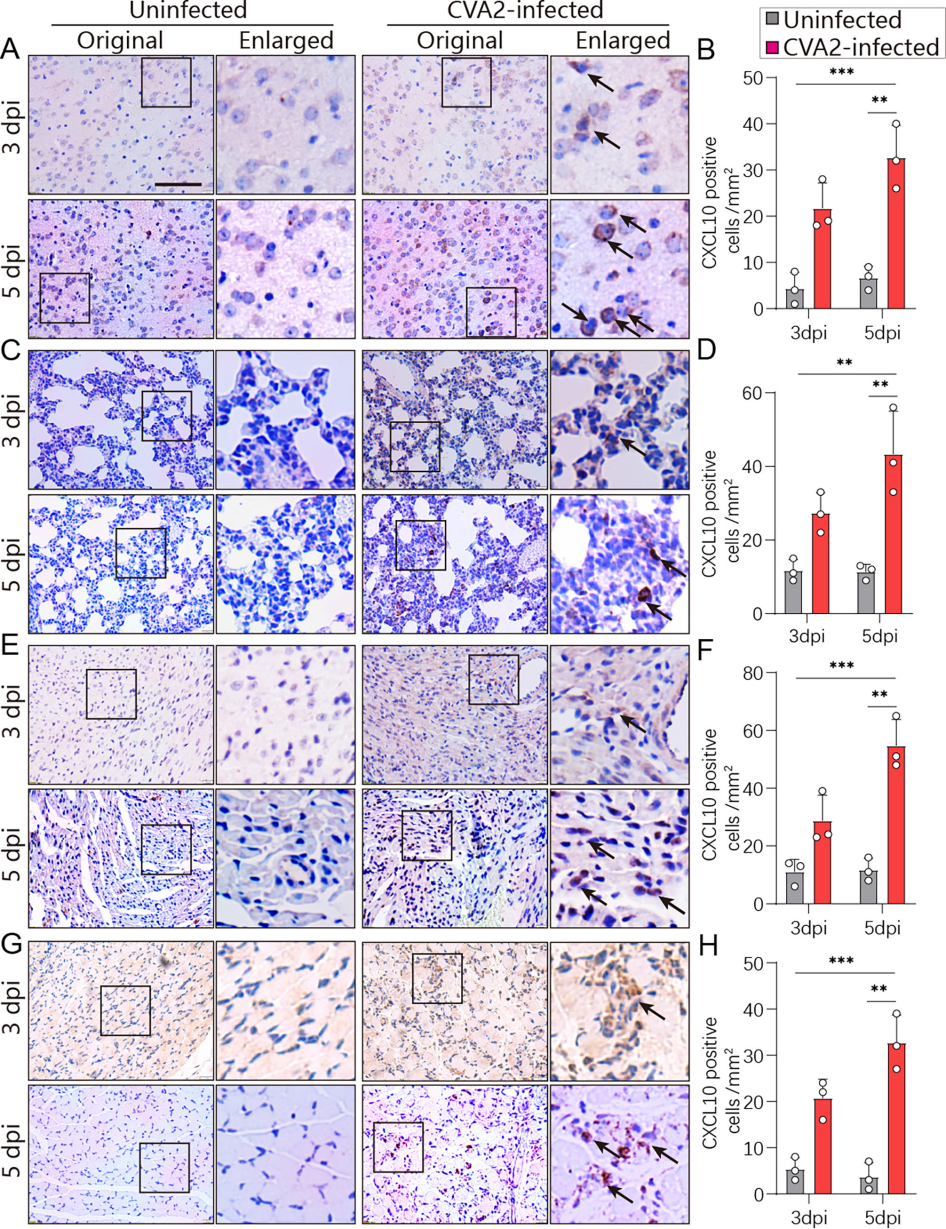
IHC staining of CXCL10 in mice infected with CVA2 at 3 dpi and 5 dpi. Black arrows indicate CXCL10 (brown). The number of CXCL10-positive cells per mm^2^ was quantified by ImageJ software. Scale bar = 50 μm. (A, B) Brains. (C, D) Lungs. (E, F) Hearts. (G, H) Skeletal muscles. *n* = 3 per group. *, *P < *0.05; **, *P < *0.01; ***, *P < *0.001.

**FIG 3:**
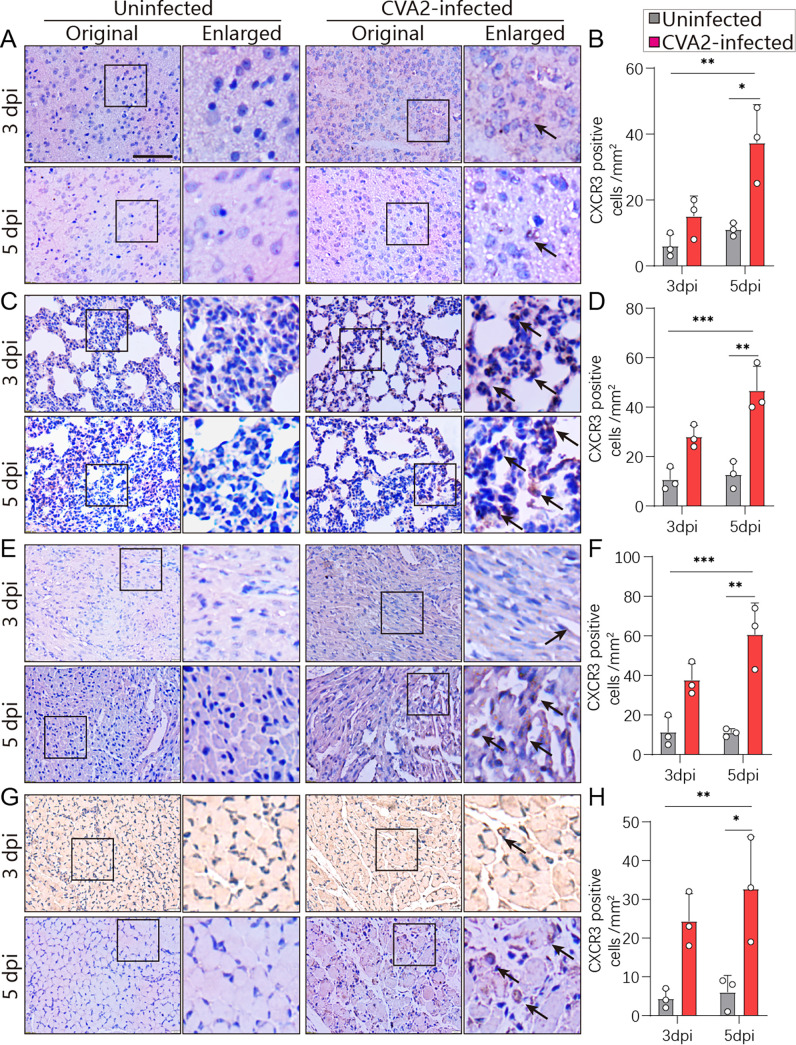
IHC staining of CXCR3 in tissue specimens of mice infected with CVA2 at 3 dpi and 5 dpi. Black arrows indicate CXCR3 (brown). The number of CXCR3-positive cells per mm^2^ was quantified by ImageJ software. Scale bar = 50 μm. (A, B) Brains. (C, D) Lungs. (E, F) Hearts. (G, H) Skeletal muscles. *n* = 3 per group. *, *P < *0.05; **, *P < *0.01; ***, *P < *0.001.

To further confirm the expression of CXCL10 and CXCR3, control- and CVA2-infected mice were sacrificed at 3 and 5 days postinfection (dpi). The organs and tissues (brains, lungs, hearts, and skeletal muscles) of mice were taken out for immunohistochemistry staining. As shown by the results in Fig. 2 and 3, CXCL10 and CXCR3 were highly expressed in the target organs and tissues from CVA2-infected mice at 3 and 5 dpi. Quantitative results showed that the expression levels of CXCL10 and CXCR3 were significantly elevated as the time after CVA2 infection increased, which agreed with the changes in transcription levels. Our results suggest that activation of the CXCL10/CXCR3 axis may contribute to CVA2 pathogenesis.

### The CXCL10/CXCR3 axis plays a crucial role in disease pathogenesis upon CVA2 infection.

**FIG 4:**
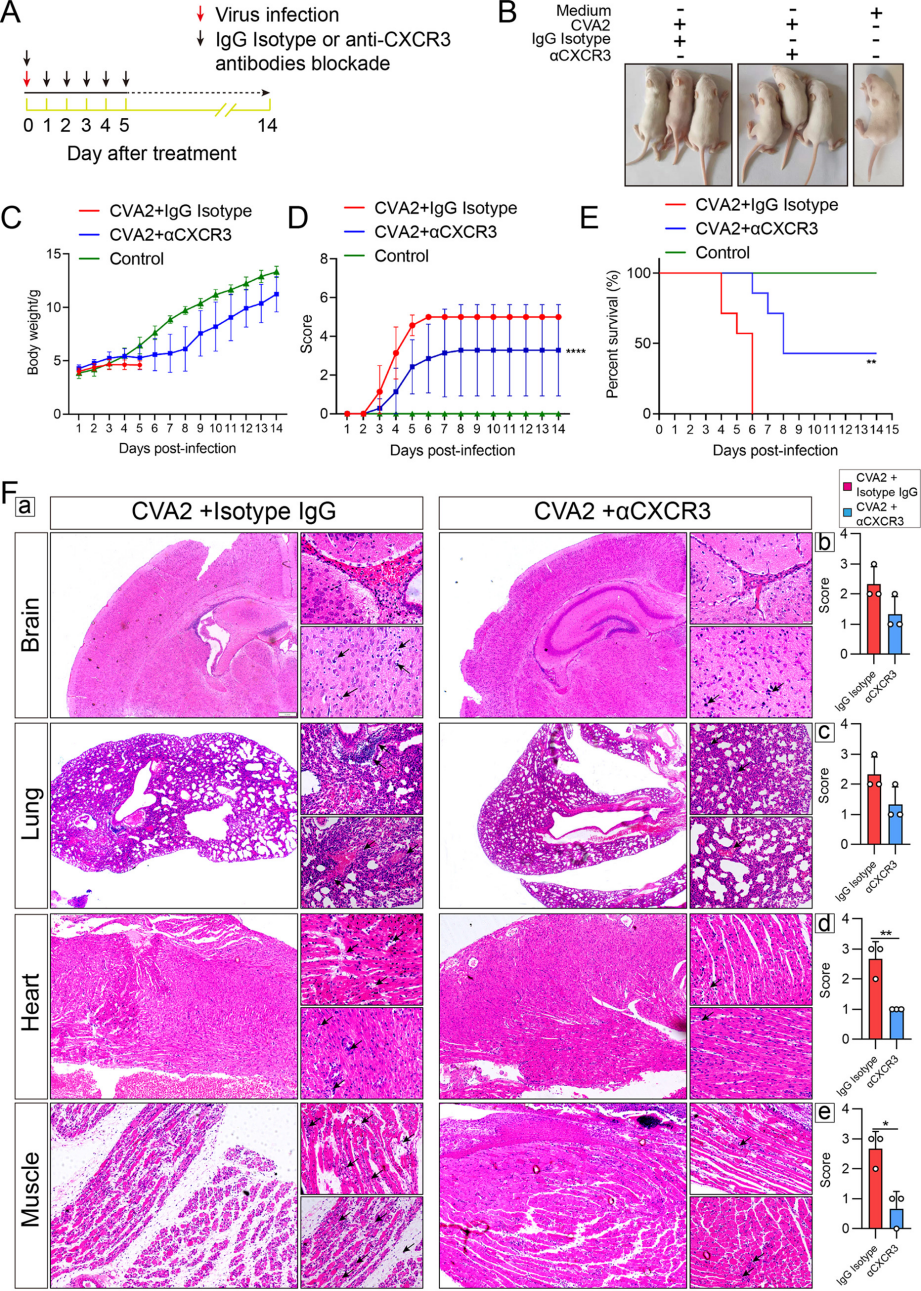
The CXCL10/CXCR3 axis plays a crucial role in disease pathogenesis upon CVA2 infection. (A) Experimental scheme showing infection with CVA2 and treatment with αCXCR3 or IgG isotype. (B) Representative clinical signs induced by CVA2 infection in IgG isotype-treated mice included weight loss, ataxia, and single or double hind limb paralysis. CVA2-infected, αCXCR3-treated mice appeared to be in relatively good health. (C to E) Body weights (C), mean clinical scores (D), and survival rates (E) of IgG-treated groups after CVA2 infection, αCXCR3-treated groups after infection, and the control group. The IgG-treated mice did not survive longer than 5 dpi, and the clinical scores on the days afterwards were carryovers from 5 dpi. *n* = 8 to 10 per group. **, *P < *0.01; ****, *P < *0.0001. (F) (a) Histopathological changes of the organs and tissues (brains, lungs, hearts, and skeletal muscles) in IgG isotype and αCXCR3 groups. Scale bar = 1 mm. (b to e) Histology scores for brains (b), lungs (c), hearts (d), and skeletal muscles (e) were evaluated by a person blinded to the treatment of groups. *n* = 3 per group. *, *P < *0.05; **, *P < *0.01.

To further assess the role of the CXCL10/CXCR3 axis in disease pathogenesis upon CVA2 infection, 5-day-old mice were injected intraperitoneally (i.p.) with CVA2 and then treated with isotype IgG or αCXCR3 (Fig. 4A). Upon CVA2 infection, the body size of IgG-treated mice was obviously reduced, whereas the body size of αCXCR3-treated mice was only slightly reduced relative to that of uninfected controls (Fig. 4B). We further found that upon CVA2 infection, IgG-treated mice displayed significantly lower body weights (Fig. 4C), much higher clinical scores (Fig. 4D), and a lower survival rate (Fig. 4E) than did mice with αCXCR3 treatment. These results indicated that CVA2-infected mice might be partially cured by αCXCR3. To determine the effect of αCXCR3 treatment on histopathological changes in CVA2-infected mice, the brains, lungs, hearts, and skeletal muscles from CVA2-infected mice given αCXCR3 or isotype control treatment were sampled at 5 dpi and histologically examined by hematoxylin and eosin (H&E) staining. We found that pathological changes in major organs were alleviated in αCXCR3-treated CVA2-infected mice, to some extent. A moderate decrease in cell damage and gliosis in brains, reductions in leakage of erythrocytes and immune cell numbers in lungs, lessened cardiomyocyte damage in hearts, and meliorative myofibrils in skeletal muscle were observed in the αCXCR3-treated mice (Fig. 4Fa). The above-described changes were likely blocked by αCXCR3 (Fig. 4Fb to Fe), and it worked effectively in hearts and muscles but only moderately improved the pathology in brains and lungs, indicating that αCXCR3 treatment alleviated CVA2-induced illness *in vivo*.

### Blockade of CXCR3 shows protective effect against CVA2 infection.

**FIG 5:**
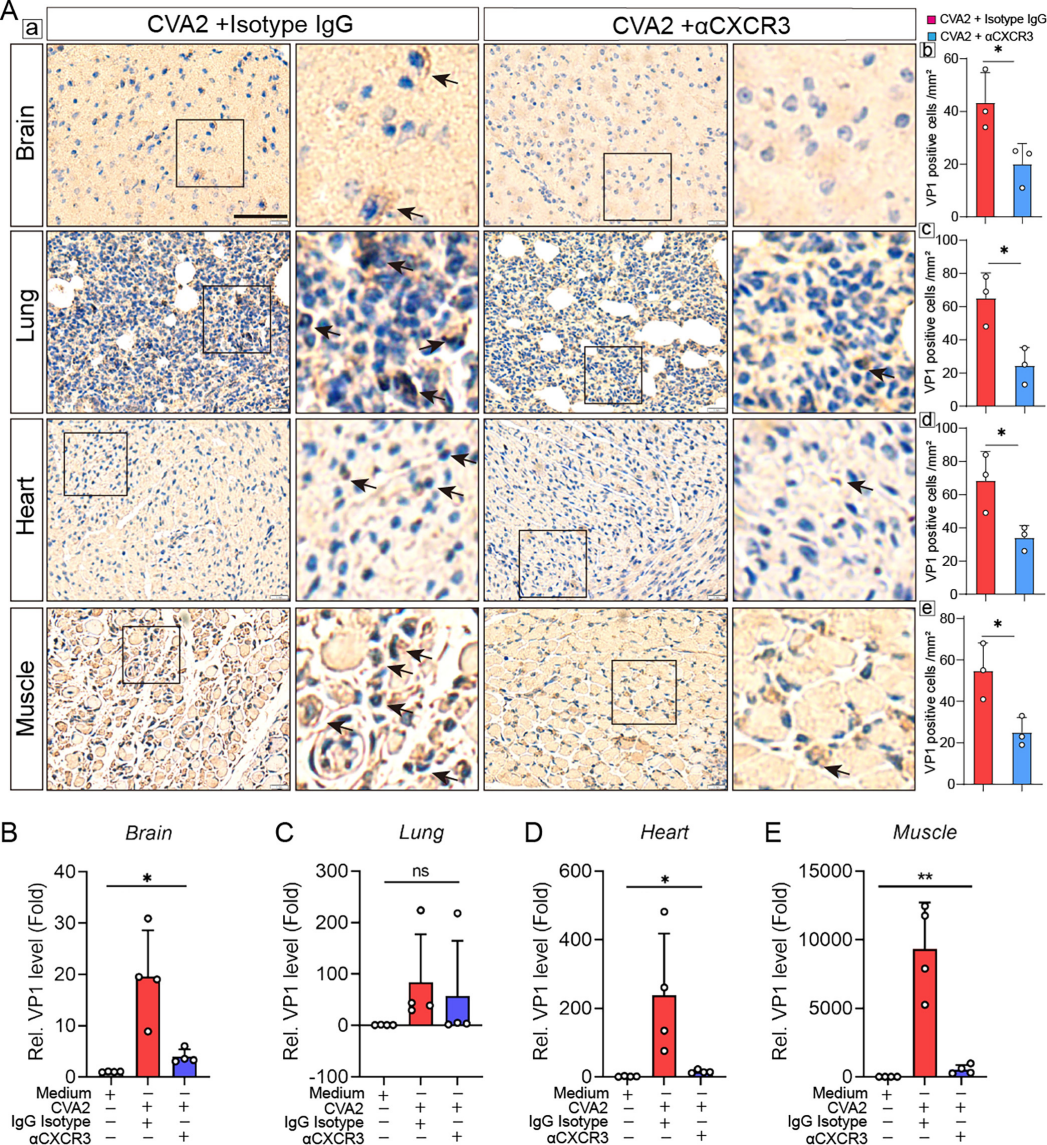
Viral distribution in the organs and tissues of IgG isotype- or αCXCR3-treated mice after CVA2 infection. (A) (a) Immunohistochemical staining of viral antigen was conducted in the slices of brains, lungs, hearts, and skeletal muscles. Black arrows indicate the locations of viral antigen. Bar = 50 μm. The number of VP1-positive cells per mm^2^ was quantified by ImageJ software. *n* = 3 per group. (b) Brains. (c) Lungs. (d) Hearts. (e) Skeletal muscles. (B to E) Relative expression of VP1 gene in brains (B), lungs (C), hearts (D), and skeletal muscles (E). *n* = 4 per group. ns, not significant; *, *P < *0.05; **, *P < *0.01.

To understand the antiviral effects of αCXCR3 treatment *in vivo*, we evaluated the viral loads in the organs and tissues (brains, lungs, hearts, and skeletal muscles) of CVA2-infected mice after αCXCR3 or isotype IgG treatment. As shown by the results in Fig. 5Aa, CVA2 antigens located in the slices of organs and tissues from αCXCR3-treated mice were dramatically decreased relative to those from IgG-treated mice after CVA2 infection. Next, we determined the transcription levels of VP1 in the organs and tissues. Our results showed that the transcription levels of VP1 in the brains (Fig. 5B), hearts (Fig. 5D), and skeletal muscles (Fig. 5E) of αCXCR3-treated mice were significantly reduced compared to the levels in IgG-treated mice after CVA2 infection. The reduced transcription levels of VP1 were most pronounced in muscles and hearts, followed by brains, and no statistical difference was found in lungs (Fig. 5B to E). These results suggest that CXCL10/CXCR3 axis activation may facilitate CVA2 replication, although it has functions in the clearance of virus, and CVA2 possibly indirectly induced the pathological damage of organs and tissues through the CXCL10/CXCR3 axis.

### Blockade of CXCR3 alleviates apoptosis and proinflammatory cytokine expression induced by CVA2 and causes alteration of inflammatory cell infiltration.

**FIG 6:**
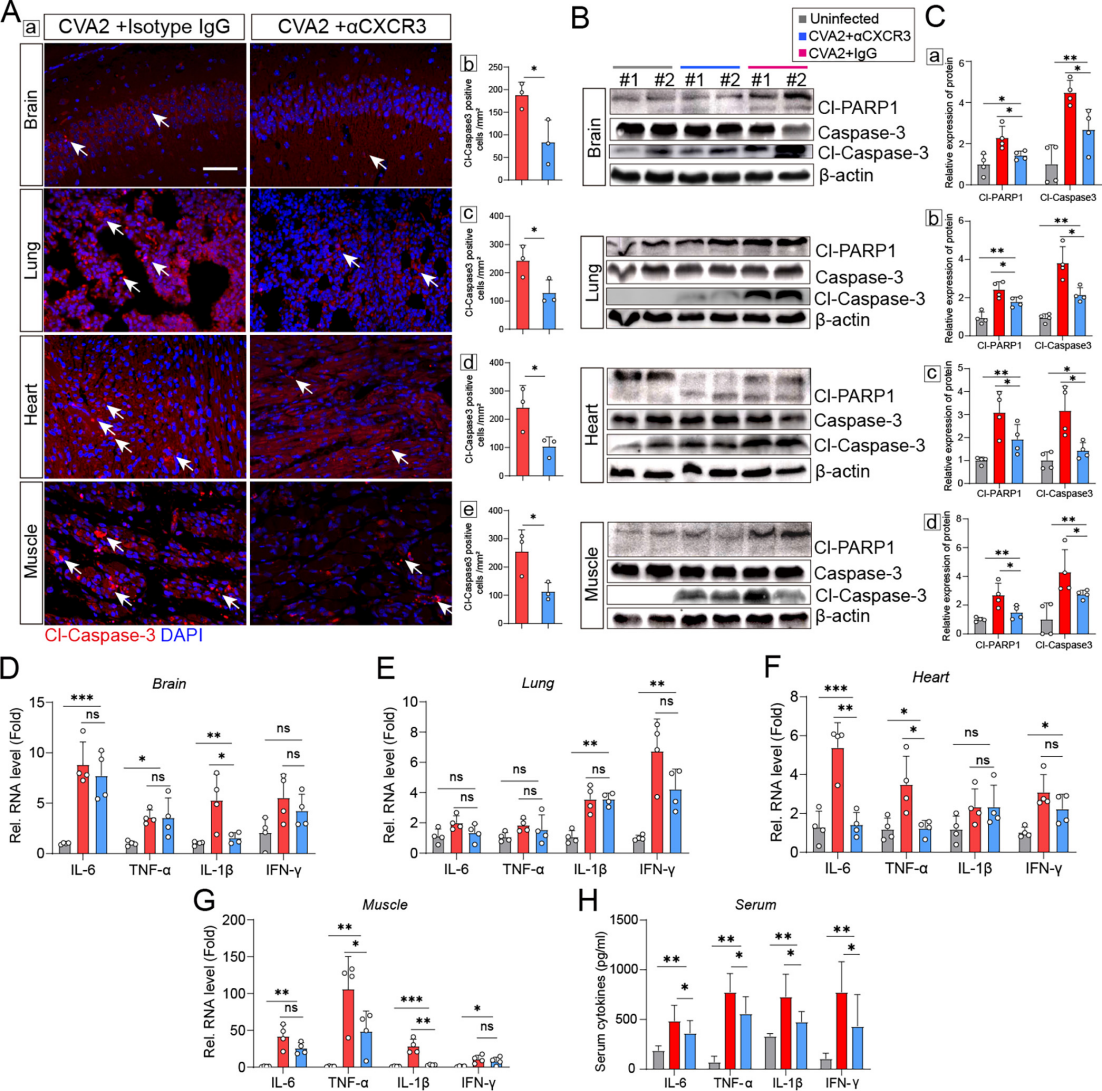
Blockade of CXCR3 alleviates apoptosis and proinflammatory cytokine expression induced by CVA2. At 5 dpi, the proteins related to apoptosis and genes related to proinflammatory cytokines were determined in IgG isotype and αCXCR3 groups. (A) Immunofluorescence staining of Cl-Caspase-3 (red) was conducted in the organs and tissues. Scale bar = 50 μm. The number of Cl-Caspase-3-positive cells per mm^2^ was quantified with ImageJ software. *n* = 3 per group. (A) (b) Brains. (c) Lungs. (d) Hearts. (e) Skeletal muscles. (B) Protein levels of Cl-Caspase-3, Cl-PARP1, and β-actin were measured by Western blotting. *n* = 2. (C) Densitometry measures for Western blots using ImageJ software in specimens from (a) brains, (b) lungs, (c) hearts, and (d) skeletal muscles. *n* = 4. (D to G) Relative expression levels of inflammatory cytokines (IL-1β, IL-6, TNF-α, and IFN-γ) were tested in brains (D), lungs (E), hearts (F), and skeletal muscles (G). *n* = 4 per group. (H) Expression levels of inflammatory cytokines in serum. *n* = 8. ns, not significant; *, *P < *0.05; **, *P < *0.01; ***, *P < *0.001.

**FIG 7:**
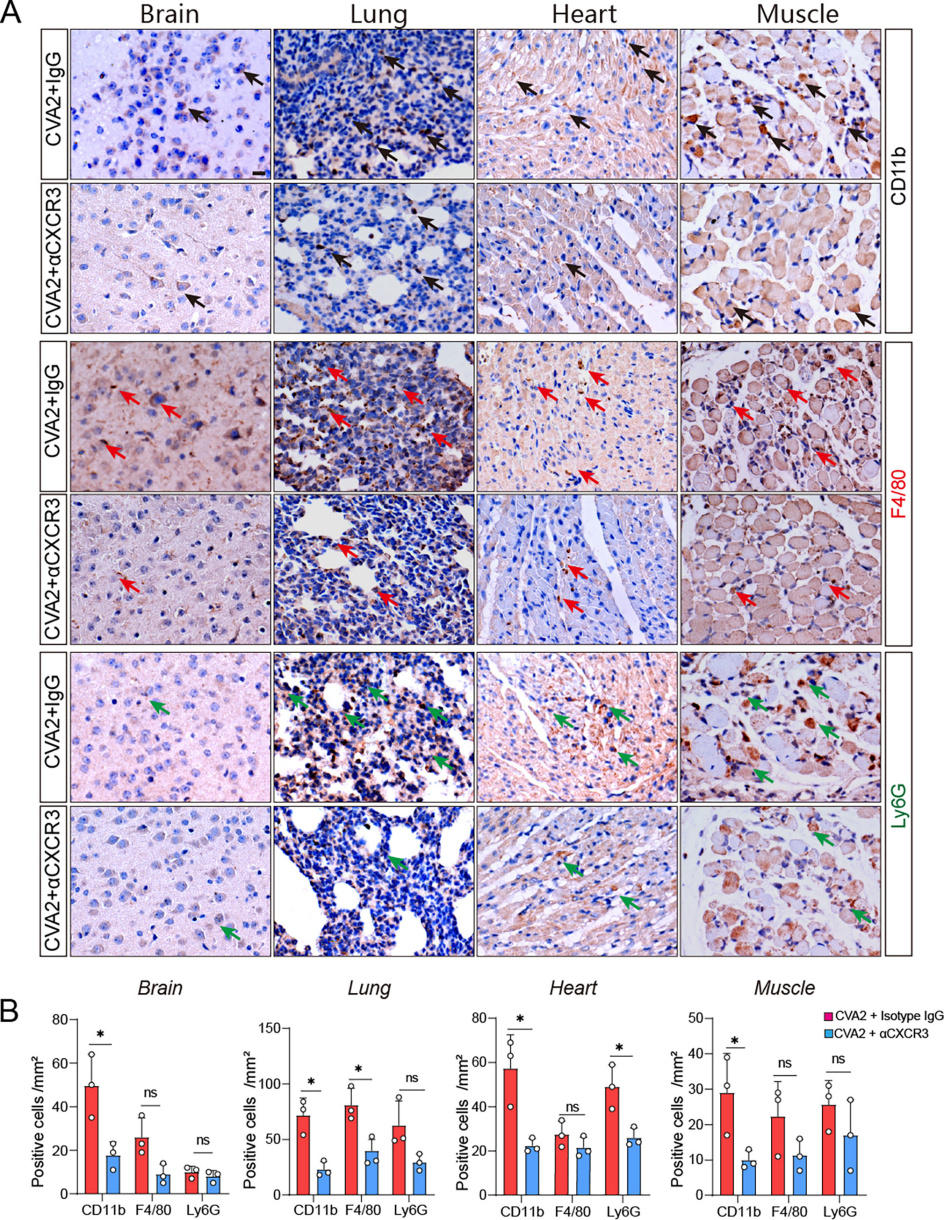
Blockade of CXCR3 causes alteration of inflammatory cell infiltration. (A) IHC staining of CD11b, F4/80, and Ly6G in organs and tissues from IgG isotype- and αCXCR3-treated mice. Black arrows mark CD11b^+^ cells (mononuclear leucocytes), red arrows mark F4/80^+^ cells (macrophages), and green arrows mark Ly6G^+^ cells (neutrophils). (B) Numbers of CD11b^+^ cells, F4/80^+^ cells, and Ly6G^+^ cells per mm^2^ in the organs and tissues of mice.

Given that the CXCL10/CXCR3 axis can mediate apoptosis by intensified leukocyte recruitment (17), we detected the activation of Caspase-3 and cleaved poly(ADP-ribose) polymerase (PARP1) (Cl-PARP1) via immunofluorescence staining and Western blotting. As shown by the images in Fig. 6A, the expression levels of cleaved Caspase-3 (Cl-Caspase-3) located in slices of the organs and tissues (brains, hearts, lungs, and skeletal muscles) from αCXCR3-treated mice were lower than the levels in IgG-treated mice after CVA2 infection. Consistently, lower expression levels of Cl-Caspase-3 and Cl-PARP1 were found in the organs and tissues from αCXCR3-treated mice (Fig. 6B). HFMD severity is thought to be associated with a cytokine storm in patients. The levels of several proinflammatory cytokines after αCXCR3 treatment were measured in mouse tissue lysates at 5 dpi. As shown by the results in Fig. 6C to F, the transcription levels of TNF-α, IL-6, IL-1β, and IFN-γ in the organs and tissues from IgG-treated mice were all significantly increased at 5 dpi, except for IL-1β in hearts. After αCXCR3 treatment, the levels of IL-1β in the brains and muscles, the levels of TNF-α in the hearts and muscles, and the levels of IL-6 in the hearts of CVA2-infected mice were significantly reduced, but the levels of IL-1β in the lungs and hearts, the levels of TNF-α in the brains and lungs, the levels of IL-6 in all organs and tissues except for hearts, and the levels of IFN-γ in all organs and tissues were not affected. Similarly, αCXCR3 treatment appeared to be most effective in the reduction of cytokines in the hearts and muscles, followed by the brains, and the least effective in the lungs. Since variations in serum cytokines are key features in HFMD severity, serum cytokine (IL-6, TNF-α, IL-1β, and IFN-γ) levels were assessed by enzyme-linked immunosorbent assay (ELISA). As shown by the results in Fig. 6G, compared with those in IgG-treated mice, lower expression levels of IL-6, TNF-α, IL-1β, and IFN-γ were detected in the αCXCR3-treated mice. Additionally, the degree of inflammatory cell infiltration was reversed by αCXCR3. As shown by the results in Fig. 7, the numbers of mononuclear leucocytes (CD11b^+^) in all of the organs and tissues, macrophages (F4/80^+^) in the lungs, and neutrophils (Ly6G^+^) in the hearts of IgG-treated mice were significantly higher than the numbers in αCXCR3-treated mice. Collectively, our data suggest that CVA2-induced apoptosis and inflammatory processes were partially reversed by αCXCR3.

## DISCUSSION

HFMD, a common communicable disease among children, has caused several large outbreaks across the Asia-Pacific region, and it represents a global public health threat. As the spectrum of disease pathogens changes, CVA2 has become one of the predominant serotypes of HFMD. Most HFMD patients present with mild to moderate symptoms, but in some patients, the disease develops into severity, with encephalitis, meningitis, cardiopulmonary failure, and even death. Due to the control of EV71 by vaccination, reports of CVA2 outbreaks and deaths have aroused widespread concern. To date, a specific antiviral treatment is still not available, and therefore, it is urgent to investigate HFMD pathogenesis, which will be beneficial for antiviral drug development. CXCL10, also known as IFN-γ-induced protein 10 (IP-10), acts through its cognate receptor CXCR3 and regulates various cells, including endothelial cells, monocytes, T cells, and dendritic cells. The activation of the CXCL10/CXCR3 axis contributes to the pathogenesis of inflammatory diseases. However, the importance of the role of the CXCL10/CXCR3 axis during CVA2 pathogenesis has not yet been investigated.

A systemic inflammatory response and central nervous system (CNS) inflammation are the main pathological features of fatal human HFMD cases (18–20). In the present study, we first screened the transcript levels of genes related to antiviral intrinsic immune factors and inflammatory processes in organs and tissues (brains, lungs, hearts, and skeletal muscles) from CVA2-infected mice. Our data suggest that the highly upregulated genes are involved in reactive oxygen species (ROS), IFN-mediated signaling pathways, IRF signaling, and NLRP3 activation-associated signaling pathways that can regulate the generation of inflammatory cytokines (21). Likewise, the proinflammatory genes encoding TNF-α, TNFR2, IL-6, and IL-1β, which can induce tissue injury, were also upregulated in multiple organs and tissues (22). Our previous study found that the activation of inflammatory pathways and proinflammatory cytokine release participated in lung and heart injury caused by CVA2 (23, 24). In the brain, the cell apoptosis mediated by the trigger of TLR7 signaling and release of IL-6 led to neural pathogenesis induced by EV71 (25), and TNF-α could prompt the blood-brain barrier (BBB) damage induced by Japanese encephalitis (26). Therefore, uncontrollable activation of both pro- and anti-inflammatory responses is the main pathophysiological feature of CVA2-induced disease *in vivo*.

Importantly, among these upregulated genes, the magnitude of the change in CXCL10 is the most obvious. Our further experiments demonstrated the activation of the CXCL10/CXCR3 axis in multiple organs and tissues of mice at 5 dpi. TLR3 and RIG-I can activate the transcription of CXCL10 through binding of NF-κB and IRF7 to CXCL10’s promoter (27). IFN-γ enhances the production of CXCL10 via the JAK/STAT pathway (28). ROS are necessary for the production of CXCL10 (29). As mentioned earlier, these upstream genes of CXCL10 were elevated in CVA2-infected mice. The activation of CXCL10/CXCR3 recruits immune cells, such as T cells, NK cells, and macrophages, to the inflamed tissue (30), regulates T cell and bone marrow progenitor maturation, and modulates adhesion molecule expression, and it inhibits angiogenesis as well (31–33). Although CXCL10/CXCR3 can clear virus via promoting NK cells and antibody responses (34), it perpetuates the autoimmune process by creating an amplification feedback loop of inflammation (35). Observational studies found that serum CXCL10 was elevated in patients with severe HFMD and CXCL10 was an important indicator of prognosis (12). Our previous population-based study suggested that significantly elevated expression of serum IFN-γ was associated with the development of HFMD severity (36). Our previous animal experiments also found an increase of serum CXCL10 in human scavenger receptor class B member 2 (SCARB2) knock-in mice after EV71 infection (37). Collectively, the activation of the CXCL10/CXCR3 axis may contribute to disease pathogenesis of mice upon EV infection.

The activation of CXCL10/CXCR3 is linked to many infectious diseases. Deficiency of CXCL10/CXCR3 or application of an CXCR3 antagonist could alleviate the symptoms or increase the survival rates of mice via reducing inflammatory cell infiltration induced by viruses (38–40). Inflammatory cells recruited by chemokines are essential for the control of infection, but tissue damage will occur when the immune response fails and uncontrolled inflammatory cells are recruited to the site of infection (41). To explore the immunopathological role of the CXCL10/CXCR3 axis in CVA2 infection, we used an anti-CXCR3 neutralizing antibody against the higher expression of CXCL10. As expected, blockade of CXCR3 showed protective effects on CVA2-infected mice. We also found that the viral loads in the organs and tissues (brains, lungs, hearts, and skeletal muscles) were decreased in αCXCR3-treated mice. These findings appear to contradict a previous study that reported that viral titers and myocardial injury were higher and more serious in CXCL10^−/−^ mice than in controls after CVB3 infection (34). Knockout gene models alone may produce the opposite of the desired result, losing the normal immune response by completely depleting the chemotaxis of one specific chemokine (34) or maintaining an otherwise excessive inflammatory response through compensatory trafficking of inflammatory cells (42). In contrast, neutralizing antibodies *in vivo* reduce the infiltration without affecting the proliferation of the associated inflammatory cells (43). We speculate that the proper activation of the CXCL10/CXCR3 axis has an antiviral effect and that proper neutralization, but not deletion, of CXCR3 exerts anti-inflammatory effects.

CXCL10/CXCR3 plays an important role in the directional movement of macrophages, NK cells, and T cells toward the inflammatory sites, and the recruited inflammatory cells further produce cytokines at the site of inflammation, which in turn contribute to the release of chemokines and finally form an inflammatory feedback loop (44). IL-6, TNF-α, IL-1β, and IFN-γ are important for inflammatory responses that further lead to tissue injury. Our results found that αCXCR3 treatment suppressed the recruitment of inflammatory cells (mononuclear leucocytes, macrophages, and neutrophils) in the multiple organs and tissues, which might further lead to the reduction of proinflammatory cytokines (45). Thus, it was apparent that the CXCL10/CXCR3 axis played a crucial role in the inflammatory response induced by CVA2. Higher expression of CXCL10 leads to apoptosis in viral encephalitis through triggering a caspase cascade (46). We found that αCXCR3 treatment inhibited the expression of the active form of Caspase-3 and PRAP1. These results suggest that CXCL10/CXCR3-mediated apoptosis aggravates the pathology of CVA2 infection, which is reversed by αCXCR3. CXCL10 is also an initial factor for the development of BBB permeability, which facilitates the infiltration of inflammatory cells (47). The neutralizing of CXCR3 decreases TNF-α-mediated BBB damage during viral infection (26). We found that αCXCR3 treatment alleviated tissue damage in the brain, as well as decreasing the spread of virus into the brain.

Based on our data, CVA2 could replicate in multiple organs and tissues in a murine model of CVA2 infection, but the viral loads were the highest in muscles, followed by hearts, and were least in the brains and lungs. Muscle is the major site of viral replication and may be the source of virus entering the CNS, which may give rise to corresponding degrees of inflammation in the four types of tissues. Meanwhile, different degrees of pathological damage in different tissues were found in CVA2-infected mice, and CVA2 caused the most severe damage to muscle and heart, followed by brains, and the least to lungs (48). Various degrees of IL-6 elevation in different tissues were detected in EV71-infected mice, and anti-IL-6 antibody therapy was most effective in muscle (49). Consistent with the different changes in inflammatory cytokines induced by EVs in different tissues, αCXCR3 could reverse the inflammatory response and improve tissue damage, appearing to be most effective in muscles and hearts, followed by brains, and least effective in lungs. We propose that the anti-CXCR3 antibody treatment alleviates inflammatory cell infiltration and further reduces inflammatory cytokine production after CVA2 infection, which finally prevents the subsequent destruction of the organs or tissues from uncontrollable cell-mediated autoimmunity (50).

In conclusion, our study presents the first evidence for the involvement of the CXCL10/CXCR3 axis in the disease pathogenesis induced by CVA2. The activation of CXCL10/CXCR3 contributes to CVA2 pathogenesis via inducing apoptosis and inflammatory cytokines, which can be reversed by an anti-CXCR3 neutralizing antibody. This study provides new insight into the pathogenesis of HFMD, which has an important guiding significance for the treatment of HFMD. A limitation in this study is that our findings are only novel for the CVA2 strain, which means infection by other EVs needs to be studied to support these observations.

## MATERIALS AND METHODS

### Ethics statement.

The study presented here was approved by the Life Science Ethics Review Committee of Zhengzhou University (permission no. ZZUIRB2020-29).

### Cell culture and viruses.

Human rhabdomyosarcoma (RD) cells and African green monkey kidney (Vero) cells cultured in Dulbecco’s modified Eagle’s medium (DMEM) (Gibco Company, New York, NY, USA) supplemented with 10% fetal bovine serum (FBS) (Gibco Company, New York, NY, USA) were purchased from the National Collection of Authenticated Cell Cultures (Shanghai, China) and incubated at 37°C with 5% CO_2_. The CVA2 strain (HN202009, accession number MT992622) used in this study was isolated from the stool of a severe case hospitalized in the First Affiliated Hospital of Xinxiang Medical University as described previously (48).

### Animal infection experiments.

The specific-pathogen-free (SPF) BALB/c mice used in this study were obtained from the Experimental Animal Center of Zhengzhou University, and all mice were housed in SPF laboratory environments in the College of Public Health, Zhengzhou University, on a 12-h-light/-dark cycle with *ad libitum* access to food and water. For *in vivo* experiments, each mouse was inoculated with a lethal dose of the CVA2 strain (10^4^ 50% tissue culture infective dose [TCID_50_]/mouse). To investigate the role of the CXCL10/CXCR3 axis in CVA2 pathogenesis, we used anti-CXCR3 neutralizing antibody (clone CXCR3-173) (Bio X Cell, Lebanon, NH, USA) (αCXCR3) and polyclonal Armenian hamster IgG isotype control antibody (Bio X Cell). Both were administered via the intraperitoneal (i.p.) route on the same day as the infection and every 24 h until 5 days postinfection (dpi). The quantity of antibodies administered was 100 μg of monoclonal antibody (MAb) per mouse. The body weight, clinical score, and survival state were recorded from day 1 to 14 after CVA2 inoculation. The grade of clinical disease was scored as follows: 0, healthy; 1, lethargic and inactive; 2, ataxic; 3, losing weight; 4, hind limb paralysis; 5, dying or dead. The control mice were inoculated with an equal volume of culture supernatants of RD cells.

### RNA extraction, reverse transcription, and qPCR.

Total RNA was extracted from organs and tissues (brains, lungs, hearts, and skeletal muscles) of mice at 3 days postinfection (dpi) and 5 dpi. The concentration of total RNA was measured using a NanoDrop ND-2000 (Thermo Fisher Scientific, Waltham, MA, USA). Reverse transcription (RT) was then performed with Hifair II 1st-strand cDNA synthesis supermix (Yeasen BioTechnologies Co., Ltd., Shanghai, China). Each cDNA was initially denatured at 95°C for 5 min and then amplified for 40 cycles of 10 s at 95°C, 30 s at 60°C using the Quantagene q225 (Kubo Technology Co., Ltd., Beijing, China). The transcription levels of relevant genes were normalized to that of the β-actin gene and were calculated by the cycle threshold (2^−ΔΔ^*^CT^*) method. The primers used for the above-described experiments are listed in Table S1 in the supplemental material.

### ELISA.

The blood from mice was collected by heart puncture, and serum was obtained after centrifugation for 10 min at 12,000 rpm. The expression levels of IL-6, TNF-α, IL-1β, and IFN-γ in serum were measured using the corresponding ELISA kits (Biolegend, San Diego, CA, USA), and the results detected by ELISA were expressed in pg/mL. The detection ranges were 26 to 2,900 pg/mL for IL-6, 36 to 2,900 pg/mL for TNF-α, 150 to 2,500 pg/mL for IL-1β, and 30 to 7,600 pg/mL for IFN-γ.

### Western blotting.

Proteins of all tissues were extracted using a protein extraction kit (Cwbio Company Ltd., Beijing, China) and mixed with an equal amount of 2× sodium dodecyl sulfate (SDS) loading buffer. Proteins were separated by 10% SDS-PAGE and transferred to polyvinylidene fluoride (PVDF) membranes. Membranes were blocked for 1 h at room temperature using 5% nonfat milk powder and incubated in primary antibodies overnight at 4°C. The primary antibodies used in this study included anti-PARP1 antibody (Wuhan Servicebio Technology Co., Wuhan, China), anti-Caspase-3 antibody, and anti-cleaved-Caspase-3 antibody (Cell Signaling Biotechnology, Inc., MA, USA). After incubation with the primary antibody, the membranes were washed 3 times, incubated with secondary antibody (Proteintech Group, Inc., Wuhan, China) for 1 h at room temperature, washed 3 times, and finally developed using the enhanced chemiluminescence (ECL) kit (Absin Bioscience, Inc., Shanghai, China). The expression levels of proteins were quantified by densitometry using Image J software.

### Histopathological examination and immunofluorescence staining.

Control mice, infected mice with administration of anti-CXCR3 neutralizing antibody, and infected mice with administration of IgG isotype control antibody were all euthanized at 5 dpi. Tissue and organ (brains, lungs, hearts, and skeletal muscles) specimens were taken out and fixed in 4% paraformaldehyde for 48 h. After fixation, paraffin-embedded lungs were cut into 5-μm sections and stained with hematoxylin and eosin (H&E). Histology scores of the tissues and organs were evaluated on a gradation of 0 to 4 with increments of 0.5 by a person blinded to the treatment of the groups. For immunofluorescence staining, the slices were stained following a standard protocol, and the number of positive stained cells per slice was quantified by Image J software.

### IHC staining.

Control mice and infected mice were all euthanized at 3 dpi and 5 dpi. Tissue and organ (brains, lungs, hearts, and skeletal muscles) specimens were taken out and fixed in 4% paraformaldehyde for 48 h. After fixation, paraffin-embedded lungs were cut into 5-μm sections and the slices were then stained with CXCL10 and CXCR3 primary antibodies (1:100, Absin Bioscience, Inc., Shanghai, China). The reaction was revealed using a biotin-streptavidin horseradish peroxidase (HRP) detection systems (ZSGB-Bio, Beijing, China). As mentioned above, the slices of the organ and tissue specimens from mice with the administration of αCXCR3 or IgG isotype control were stained with anti-CVA2 VP1 mouse monoclonal antibody (prepared in our own laboratory), anti-CD11b antibody, anti-F4/80 antibody, and anti-Ly6G antibody (Wuhan Servicebio Technology Co., Wuhan, China), and the reactions were revealed using a biotin-streptavidin HRP detection system (ZSGB-Bio, Beijing, China). The number of positive stained cells per slice was quantified by using ImageJ software.

### Statistical analysis.

Statistical analysis was performed with GraphPad Prism version 8.3 (GraphPad 8.3 Software, San Diego, CA, USA). The differences in the survival rates of treated versus control mice were assessed with the Mantel-Cox log rank test, and survival curves were plotted using the Kaplan-Meier method. The results were expressed as the mean values ± standard deviations (SD). Differences in gene expression, clinical scores, and viral loads were assessed using the Mann-Whitney test or one-way analysis of variance (ANOVA). A *P* value of less than 0.05 was regarded as significant.

### Data availability.

qPCR data were uploaded into the gene expression omnibus (GEO) database (https://www.ncbi.nlm.nih.gov/geo/) (accession no. GSE189549).

## References

[1] Liu Z, Wang S, Yang R, Ou X. 2014. A case-control study of risk factors for severe hand-foot-mouth disease in Yuxi, China, 2010–2012. Virol Sin 29:123–125. doi:10.1007/s12250-014-3384-3. 24643937 PMC8206253

[2] Li Y, Chang Z, Wu P, Liao Q, Liu F, Zheng Y, Luo L, Zhou Y, Chen Q, Yu S, Guo C, Chen Z, Long L, Zhao S, Yang B, Yu H, Cowling BJ. 2018. Emerging enteroviruses causing hand, foot and mouth disease, China, 2010–2016. Emerg Infect Dis 24:1902–1906. doi:10.3201/eid2410.171953. 30226172 PMC6154135

[3] Yen TY, Huang YP, Hsu YL, Chang YT, Lin HC, Wu HS, Hwang KP. 2017. A case of recombinant coxsackievirus A2 infection with neurological complications in Taiwan. J Microbiol Immunol Infect 50:928–930. doi:10.1016/j.jmii.2016.08.012. 28082064

[4] Yip CC, Lau SK, Woo PC, Wong SS, Tsang TH, Lo JY, Lam WK, Tsang CC, Chan KH, Yuen KY. 2013. Recombinant coxsackievirus A2 and deaths of children, Hong Kong, 2012. Emerg Infect Dis 19:1285–1288. doi:10.3201/eid1908.121498. 23876841 PMC3739500

[5] Hu YF, Yang F, Du J, Dong J, Zhang T, Wu ZQ, Xue Y, Jin Q. 2011. Complete genome analysis of coxsackievirus A2, A4, A5, and A10 strains isolated from hand, foot, and mouth disease patients in China revealing frequent recombination of human enterovirus A. J Clin Microbiol 49:2426–2434. doi:10.1128/JCM.00007-11. 21543560 PMC3147834

[6] Baek K, Yeo S, Lee B, Park K, Song J, Yu J, Rheem I, Kim J, Hwang S, Choi Y, Cheon D, Park J. 2011. Epidemics of enterovirus infection in Chungnam Korea, 2008 and 2009. Virol J 8:297. doi:10.1186/1743-422X-8-297. 21668960 PMC3130694

[7] Chansaenroj J, Auphimai C, Puenpa J, Mauleekoonphairoj J, Wanlapakorn N, Vuthitanachot V, Vongpunsawad S, Poovorawan Y. 2017. High prevalence of coxsackievirus A2 in children with herpangina in Thailand in 2015. Virusdisease 28:111–114. doi:10.1007/s13337-017-0366-8. 28466062 PMC5377860

[8] Sousa IP, Jr, Oliveira MLA, Burlandy FM, Machado RS, Oliveira SS, Tavares FN, Gomes-Neto F, da Costa EV, da Silva EE. 2020. Molecular characterization and epidemiological aspects of non-polio enteroviruses isolated from acute flaccid paralysis in Brazil: a historical series (2005–2017). Emerg Microbes Infect 9:2536–2546. doi:10.1080/22221751.2020.1850181. 33179584 PMC7717866

[9] Wong KT, Munisamy B, Ong KC, Kojima H, Noriyo N, Chua KB, Ong BB, Nagashima K. 2008. The distribution of inflammation and virus in human enterovirus 71 encephalomyelitis suggests possible viral spread by neural pathways. J Neuropathol Exp Neurol 67:162–169. doi:10.1097/nen.0b013e318163a990. 18219253

[10] Wang SM, Lei HY, Yu CK, Wang JR, Su IJ, Liu CC. 2008. Acute chemokine response in the blood and cerebrospinal fluid of children with enterovirus 71-associated brainstem encephalitis. J Infect Dis 198:1002–1006. doi:10.1086/591462. 18710325

[11] Lin TY, Hsia SH, Huang YC, Wu CT, Chang LY. 2003. Proinflammatory cytokine reactions in enterovirus 71 infections of the central nervous system. Clin Infect Dis 36:269–274. doi:10.1086/345905. 12539066

[12] Sun Z, Li W, Xu J, Ren K, Gao F, Jiang Z, Ji F, Pan D. 2020. Proteomic analysis of cerebrospinal fluid in children with acute enterovirus-associated meningoencephalitis identifies dysregulated host processes and potential biomarkers. J Proteome Res 19:3487–3498. doi:10.1021/acs.jproteome.0c00307. 32678604

[13] Engel MA, Neurath MF. 2010. Anticancer properties of the IL-12 family—focus on colorectal cancer. Curr Med Chem 17:3303–3308. doi:10.2174/092986710793176366. 20712574

[14] Antonelli A, Ferri C, Ferrari SM, Colaci M, Fallahi P. 2008. Immunopathogenesis of HCV-related endocrine manifestations in chronic hepatitis and mixed cryoglobulinemia. Autoimmun Rev 8:18–23. doi:10.1016/j.autrev.2008.07.017. 18708169

[15] Lee EY, Lee ZH, Song YW. 2009. CXCL10 and autoimmune diseases. Autoimmun Rev 8:379–383. doi:10.1016/j.autrev.2008.12.002. 19105984

[16] Liu M, Guo S, Hibbert JM, Jain V, Singh N, Wilson NO, Stiles JK. 2011. CXCL10/IP-10 in infectious diseases pathogenesis and potential therapeutic implications. Cytokine Growth Factor Rev 22:121–130. doi:10.1016/j.cytogfr.2011.06.001. 21802343 PMC3203691

[17] Ha Y, Liu H, Zhu S, Yi P, Liu W, Nathanson J, Kayed R, Loucas B, Sun J, Frishman LJ, Motamedi M, Zhang W. 2017. Critical role of the CXCL10/C-X-C chemokine receptor 3 axis in promoting leukocyte recruitment and neuronal injury during traumatic optic neuropathy induced by optic nerve crush. Am J Pathol 187:352–365. doi:10.1016/j.ajpath.2016.10.009. 27960090 PMC5389365

[18] Cox JA, Hiscox JA, Solomon T, Ooi MH, Ng LFP. 2017. Immunopathogenesis and virus-host interactions of enterovirus 71 in patients with hand, foot and mouth disease. Front Microbiol 8:2249. doi:10.3389/fmicb.2017.02249. 29238324 PMC5713468

[19] Jin Y, Zhang C, Zhang R, Ren J, Chen S, Sui M, Zhou G, Dang D, Zhu J, Feng H, Xi Y, Yang H, Duan G. 2017. Pulmonary edema following central nervous system lesions induced by a non- mouse-adapted EV71 strain in neonatal BALB/c mice. Virol J 14:243. doi:10.1186/s12985-017-0911-5. 29282065 PMC5745784

[20] Solomon T, Lewthwaite P, Perera D, Cardosa MJ, McMinn P, Ooi MH. 2010. Virology, epidemiology, pathogenesis, and control of enterovirus 71. Lancet Infect Dis 10:778–790. doi:10.1016/S1473-3099(10)70194-8. 20961813

[21] Jin Y, Zhang R, Wu W, Duan G. 2018. Antiviral and inflammatory cellular signaling associated with enterovirus 71 infection. Viruses 10:155. doi:10.3390/v10040155. PMC5923449 29597291

[22] Marchant DJ, Boyd JH, Lin DC, Granville DJ, Garmaroudi FS, McManus BM. 2012. Inflammation in myocardial diseases. Circ Res 110:126–144. doi:10.1161/CIRCRESAHA.111.243170. 22223210

[23] Ji W, Hu Q, Zhang M, Zhang C, Chen C, Yan Y, Zhang X, Chen S, Tao L, Zhang W, Jin Y, Duan G. 2021. The disruption of the endothelial barrier contributes to acute lung injury induced by coxsackievirus A2 infection in mice. Int J Mol Sci 22:9895. doi:10.3390/ijms22189895. 34576058 PMC8467819

[24] Ji W, Zhu P, Liang R, Zhang L, Zhang Y, Wang Y, Zhang W, Tao L, Chen S, Yang H, Jin Y, Duan G. 2021. Coxsackievirus A2 leads to heart injury in a neonatal mouse model. Viruses 13:1588. doi:10.3390/v13081588. 34452454 PMC8402683

[25] Luo Z, Su R, Wang W, Liang Y, Zeng X, Shereen MA, Bashir N, Zhang Q, Zhao L, Wu K, Liu Y, Wu J. 2019. EV71 infection induces neurodegeneration via activating TLR7 signaling and IL-6 production. PLoS Pathog 15:e1008142. doi:10.1371/journal.ppat.1008142. 31730654 PMC6932824

[26] Wang K, Wang H, Lou W, Ma L, Li Y, Zhang N, Wang C, Li F, Awais M, Cao S, She R, Fu ZF, Cui M. 2018. IP-10 promotes blood-brain barrier damage by inducing tumor necrosis factor alpha production in Japanese encephalitis. Front Immunol 9:1148. doi:10.3389/fimmu.2018.01148. 29910805 PMC5992377

[27] Zhang Y, Liu B, Ma Y, Yi J, Zhang C, Zhang Y, Xu Z, Wang J, Yang K, Yang A, Zhuang R, Jin B. 2014. Hantaan virus infection induces CXCL10 expression through TLR3, RIG-I, and MDA-5 pathways correlated with the disease severity. Mediators Inflamm 2014:697837. doi:10.1155/2014/697837. 24701034 PMC3950924

[28] Qi XF, Kim DH, Yoon YS, Jin D, Huang XZ, Li JH, Deung YK, Lee KJ. 2009. Essential involvement of cross-talk between IFN-gamma and TNF-alpha in CXCL10 production in human THP-1 monocytes. J Cell Physiol 220:690–697. doi:10.1002/jcp.21815. 19472212

[29] You N, Li J, Huang X, Wu K, Tang Y, Wang L, Li H, Mi N, Zheng L. 2018. COMMD7 activates CXCL10 production by regulating NF-κB and the production of reactive oxygen species. Mol Med Rep 17:6784–6788. doi:10.3892/mmr.2018.8706. 29532873

[30] Singh UP, Singh S, Singh R, Cong Y, Taub DD, Lillard JW, Jr. 2008. CXCL10-producing mucosal CD4+ T cells, NK cells, and NKT cells are associated with chronic colitis in IL-10(-/-) mice, which can be abrogated by anti-CXCL10 antibody inhibition. J Interferon Cytokine Res 28:31–43. doi:10.1089/jir.2007.0059. 18370870 PMC2435497

[31] Gautam SC, Pindolia KR, Noth CJ, Janakiraman N, Xu YX, Chapman RA. 1995. Chemokine gene expression in bone marrow stromal cells: downregulation with sodium salicylate. Blood 86:2541–2550. doi:10.1182/blood.V86.7.2541.2541. 7670099

[32] Taub DD, Lloyd AR, Conlon K, Wang JM, Ortaldo JR, Harada A, Matsushima K, Kelvin DJ, Oppenheim JJ. 1993. Recombinant human interferon-inducible protein 10 is a chemoattractant for human monocytes and T lymphocytes and promotes T cell adhesion to endothelial cells. J Exp Med 177:1809–1814. doi:10.1084/jem.177.6.1809. 8496693 PMC2191047

[33] Strieter RM, Polverini PJ, Kunkel SL, Arenberg DA, Burdick MD, Kasper J, Dzuiba J, Van Damme J, Walz A, Marriott D. 1995. The functional role of the ELR motif in CXC chemokine-mediated angiogenesis. J Biol Chem 270:27348–27357. doi:10.1074/jbc.270.45.27348. 7592998

[34] Yuan J, Liu Z, Lim T, Zhang H, He J, Walker E, Shier C, Wang Y, Su Y, Sall A, McManus B, Yang D. 2009. CXCL10 inhibits viral replication through recruitment of natural killer cells in coxsackievirus B3-induced myocarditis. Circ Res 104:628–638. doi:10.1161/CIRCRESAHA.108.192179. 19168435

[35] Antonelli A, Ferrari SM, Giuggioli D, Ferrannini E, Ferri C, Fallahi P. 2014. Chemokine (C-X-C motif) ligand (CXCL)10 in autoimmune diseases. Autoimmun Rev 13:272–280. doi:10.1016/j.autrev.2013.10.010. 24189283

[36] Duan G, Yang H, Shi L, Sun W, Sui M, Zhang R, Wang X, Wang F, Zhang W, Xi Y, Fan Q. 2014. Serum inflammatory cytokine levels correlate with hand-foot-mouth disease severity: a nested serial case-control study. PLoS One 9:e112676. doi:10.1371/journal.pone.0112676. 25391156 PMC4229228

[37] Jin Y, Sun T, Zhou G, Li D, Chen S, Zhang W, Li X, Zhang R, Yang H, Duan G. 2021. Pathogenesis study of enterovirus 71 using a novel human SCARB2 knock-in mouse model. mSphere 6:e01048-20. doi:10.1128/mSphere.01048-20. 33692197 PMC8546711

[38] Ichikawa A, Kuba K, Morita M, Chida S, Tezuka H, Hara H, Sasaki T, Ohteki T, Ranieri VM, dos Santos CC, Kawaoka Y, Akira S, Luster AD, Lu B, Penninger JM, Uhlig S, Slutsky AS, Imai Y. 2013. CXCL10-CXCR3 enhances the development of neutrophil-mediated fulminant lung injury of viral and nonviral origin. Am J Respir Crit Care Med 187:65–77. doi:10.1164/rccm.201203-0508OC. 23144331 PMC3927876

[39] Fadel SA, Bromley SK, Medoff BD, Luster AD. 2008. CXCR3-deficiency protects influenza-infected CCR5-deficient mice from mortality. Eur J Immunol 38:3376–3387. doi:10.1002/eji.200838628. 19039768 PMC2749081

[40] Ferrari SM, Fallahi P, Ruffilli I, Elia G, Ragusa F, Paparo SR, Patrizio A, Mazzi V, Colaci M, Giuggioli D, Ferri C, Antonelli A. 2019. Immunomodulation of CXCL10 secretion by hepatitis C virus: could CXCL10 be a prognostic marker of chronic hepatitis C? J Immunol Res 2019:5878960. doi:10.1155/2019/5878960. 31485460 PMC6702819

[41] Christensen JE, Simonsen S, Fenger C, Sørensen MR, Moos T, Christensen JP, Finsen B, Thomsen AR. 2009. Fulminant lymphocytic choriomeningitis virus-induced inflammation of the CNS involves a cytokine-chemokine-cytokine-chemokine cascade. J Immunol 182:1079–1087. doi:10.4049/jimmunol.182.2.1079. 19124751

[42] Kwun J, Hazinedaroglu SM, Schadde E, Kayaoglu HA, Fechner J, Hu HZ, Roenneburg D, Torrealba J, Shiao L, Hong X, Peng R, Szewczyk JW, Sullivan KA, DeMartino J, Knechtle SJ. 2008. Unaltered graft survival and intragraft lymphocytes infiltration in the cardiac allograft of Cxcr3^−/−^ mouse recipients. Am J Transplant 8:1593–1603. doi:10.1111/j.1600-6143.2008.02250.x. 18476975

[43] He S, Cao Q, Qiu Y, Mi J, Zhang JZ, Jin M, Ge H, Emerson SG, Zhang Y, Zhang Y. 2008. A new approach to the blocking of alloreactive T cell-mediated graft-versus-host disease by in vivo administration of anti-CXCR3 neutralizing antibody. J Immunol 181:7581–7592. doi:10.4049/jimmunol.181.11.7581. 19017947

[44] Callahan V, Hawks S, Crawford MA, Lehman CW, Morrison HA, Ivester HM, Akhrymuk I, Boghdeh N, Flor R, Finkielstein CV, Allen IC, Weger-Lucarelli J, Duggal N, Hughes MA, Kehn-Hall K. 2021. The pro-inflammatory chemokines CXCL9, CXCL10 and CXCL11 are upregulated following SARS-CoV-2 infection in an AKT-dependent manner. Viruses 13:1062. doi:10.3390/v13061062. 34205098 PMC8226769

[45] Han J, Wang Y, Gan X, Song J, Sun P, Dong XP. 2014. Serum cytokine profiles of children with human enterovirus 71-associated hand, foot, and mouth disease. J Med Virol 86:1377–1385. doi:10.1002/jmv.23929. 24619468

[46] Sui Y, Potula R, Dhillon N, Pinson D, Li S, Nath A, Anderson C, Turchan J, Kolson D, Narayan O, Buch S. 2004. Neuronal apoptosis is mediated by CXCL10 overexpression in simian human immunodeficiency virus encephalitis. Am J Pathol 164:1557–1566. doi:10.1016/S0002-9440(10)63714-5. 15111302 PMC1615658

[47] Chai Q, She R, Huang Y, Fu ZF. 2015. Expression of neuronal CXCL10 induced by rabies virus infection initiates infiltration of inflammatory cells, production of chemokines and cytokines, and enhancement of blood-brain barrier permeability. J Virol 89:870–876. doi:10.1128/JVI.02154-14. 25339777 PMC4301165

[48] Ji W, Qin L, Tao L, Zhu P, Liang R, Zhou G, Chen S, Zhang W, Yang H, Duan G, Jin Y. 2021. Neonatal murine model of coxsackievirus A2 infection for the evaluation of antiviral therapeutics and vaccination. Front Microbiol 12:658093. doi:10.3389/fmicb.2021.658093. 34122374 PMC8192712

[49] Khong WX, Foo DG, Trasti SL, Tan EL, Alonso S. 2011. Sustained high levels of interleukin-6 contribute to the pathogenesis of enterovirus 71 in a neonate mouse model. J Virol 85:3067–3076. doi:10.1128/JVI.01779-10. 21228224 PMC3067852

[50] Tanaka S, Nishida Y, Aida K, Maruyama T, Shimada A, Suzuki M, Shimura H, Takizawa S, Takahashi M, Akiyama D, Arai-Yamashita S, Furuya F, Kawaguchi A, Kaneshige M, Katoh R, Endo T, Kobayashi T. 2009. Enterovirus infection, CXC chemokine ligand 10 (CXCL10), and CXCR3 circuit: a mechanism of accelerated beta-cell failure in fulminant type 1 diabetes. Diabetes 58:2285–2291. doi:10.2337/db09-0091. 19641142 PMC2750208

