# Supplementary material for: The CXCL10/CXCR3 Axis Promotes Disease Pathogenesis in Mice upon CVA2 Infection
Source: Microbiol Spectr. 2022 May 23;10(3):e02307-21. doi: 10.1128/spectrum.02307-21 (PMC9241849; doi:10.1128/spectrum.02307-21)
Supplement: SUPPLEMENTAL FILE 1 — Table 1, Fig. S1, Fig. S2. Download spectrum.02307-21-s001.pdf, PDF file, 1.1 MB [file spectrum.02307-21-s001.pdf]

Table 1
Primers used in this study.

Gene	Forward (5’->3’)	Reverse (5’->3’)
CYBA	TGCCAGTGTGATCTATCTGCT	TCGGCTTCTTTCGGACCTCT
CYBB	CCTCTACCAAAACCATTCGGAG	CTGTCCACGTACAATTCGTTCA
iNOS	ACATCGACCCGTCCACAGTAT	CAGAGGGGTAGGCTTGTCTC
NRF2	TAGATGACCATGAGTCGCTTGC	GCCAAACTTGCTCCATGTCC
GPX1	GTTTGAGAAGTGCGAAGTGAAT	CGGAGACCAAATGATGTACTTG
HMOX1	CACAGCACTATGTAAAGCGTCT	GTAGCGGGTATATGCGTGGG
CCL4	TTCCTGCTGTTTCTCTTACACCT	CTGTCTGCCTCTTTTGGTCAG
CCL5	GTATTTCTACACCAGCAGCAAG	TCTTGAACCCACTTCTTCTCTG
CCR1	ATCCTGTTGACGATTGACAGAT	TGATGCCAAAAGTAACAGTTCG
CCR2	ATCCACGGCATACTATCAACATC	CAAGGCTCACCATCATCGTAG
CX3CR1	GAGTATGACGATTCTGCTGAGG	CAGACCGAACGTGAAGACGAG
CXCL9	GGAGTTCGAGGAACCCTAGTG	GGGATTTGTAGTGGATCGTGC
CXCL10	CCAAGTGCTGCCGTCATTTTC	GGCTCGCAGGGATGATTTCAA
CXCL11	TGTAATTTACCCGAGTAACGGC	CACCTTTGTCGTTTATGAGCCTT
CXCR3	GGTTAGTGAACGTCAAGTGCT	CCCCATAATCGTAGGGAGAGGT
MCP1	TAAAAACCTGGATCGGAACCAAA	GCATTAGCTTCAGATTTACGGGT
TNF	CCTGTAGCCCACGTCGTAG	GGGAGTAGACAAGGTACAACCC
TNFR1	CCGGGAGAAGAGGGATAGCTT	TCGGACAGTCACTCACCAAGT
TNFR2	CCAAGGACACTCTACGTATCTC	TGATGTCACTCCAACAATCAGA
IL-1α	TTGAAGACCTAAAGAACTGTTACAGTGAA	GCCATAGCTTGCATCATAGAAGG
IL-1 β	GAAATGCCACCTTTTGACAGTG	TGGATGCTCTCATCAGGACAG
IL-16	TATGTCCACAACCTTCCCTAAC	CTTGAGTATCCTCGGATTGTGT
IL-18	AGGACAAAGAAAGCCGCCTC	TCATTTCCTTGAAGTTGACGCAAGAGT
BAFF	ACGCCGGACACTGGACATA	AGACTCACTAGACCCACCAGG
GMCSF	TTCAAGAAGCTAACATGTGTGC	GGTAACTTGTGTTTCACAGTCC
CSF1	GTGTCAGAACACTGTAGCCAC	TCAAAGGCAATCTGGCATGAAG
SPP1	AGCAAGAAACTCTTCCAAGCAA	GTGAGATTCGTCAGATTCATCCG
IL-6	CTGCAAGAGACTTCCATCCAG	AGTGGTATAGACAGGTCTGTTGG
IL-10	CTTACTGACTGGCATGAGGATCA	GCAGCTCTAGGAGCATGTGG
TGFβ1	CTTCAATACGTCAGACATTCGGG	GTAACGCCAGGAATTGTTGCTA
GBP1	TAGAGTGGATACAGGAGGACCA	GCGTTCTCCATACAGGGTAGTT
GBP2	GAGCTGTGTGGTGAATTTGTAG	CACAGAGTCTTGACAGAAGAGT
GBP4	ATGGTGATTCCCTTGTGGAAAG	AAGGAGTGATAAAACGCTGCTT
GBP5	CTGAACTCAGATTTTGTGCAGGA	CATCGACATAAGTCAGCACCAG
NLRP3	TCTTCTCAAGTCTAAGCACCAAC	ACAGCAATCTGATTCCAAAGTC
Caspase1	AGAGGATTTCTTAACGGATGCA	TCACAAGACCAGGCATATTCTT
COX2	TGCACTATGGTTACAAAAGCTGG	TCAGGAAGCTCCTTATTTCCCTT
CD68	GAAATGTCACAGTTCACACCAG	GGATCTTGGACTAGTAGCAGTG
MSR1	GAATTCACTGGATGCAATCTCC	TGTTGCTTTGCTGTAGATTCAC
MRC1	CCTATGAAAATTGGGCTTACGG	CTGACAAATCCAGTTGTTGAGG
CD163	GGACATGAGTCCCATCTTTCAC	AGCTCCACTCTGCCCTCACAC

CXCL16	AAACATTTGCCTCAAGCCAGT	GTTTCTCATTTGCCTCAGCCT
CD40	CTGTCTGTACCTGTAAGGAAGGA	AGACGGTATCAGTGGTCTCAG
CD86	CGAGCACTATTTGGGCACAGAG	TTTCCAGAACACACACAACGGTC
Cathepsin S	AAGCGGTGTCTATGACGACCC	GAGTCCCATAGCCAACCACAAG
CIITA	CCTCTTCTCTGCAGCTATAGTC	TGTAAGTGTACAAGGTAGCTGG
FRX5	GACAACGACAAGCTGTACCTC	TCGATAGGCATCATAGACGCTC
IFN β	TGGGTGGAATGAGACTATTGTTG	CTCCCACGTCAATCTTTCCTC
IRF1	GGCCGATACAAAGCAGGAGAA	GGAGTTCATGGCACAACGGA
IRF3	CTGACAATAGCAAGGACCCTTA	AGGCCATCAAATAACTTCGGTA
IRF4	AAAGGCAAGTTCCGAGAAGGG	CTCGACCAATTCCTCAAAGTCA
IRF7	CCCCAGCCGGTGATCTTTC	CACAGTGACGGTCCTCGAAG
IRF8	AGACGAGGTTACGCTGTGC	CTCCTCTTGGTCATACCCATGTA
IRF9	GATGTTGCTGAACCCTACAAAG	AGGTGACACACAACTGATACTT
AP1	TTCCTCCAGTCCGAGAGCG	TGAGAAGGTCCGAGTTCTTGG
IFNγR1	GTGGAGCTTTGACGAGCACT	ATTCCCAGCATACGACAGGGT
JAK2	CTTGTGGTATTACGCCTGTGT	TGCCTGGTTGACTCGTCTATG
STAT1	GCTGCCTATGATGTCTCGTTT	TGCTTTTCCGTATGTTGTGCT
STAT2	CTGAAGGACGAACAGGATGTC	CAGGGTGGTTAATCGGCCAA
IFIT1	ATCGCGTAGACAAAGCTCTTC	GTTTCGGGATGTCCTCAGTTG
IFITM1	GACAGCCACCACAATCAACAT	CCCAGGCAGCAGAAGTTCAT
OAS1	GAGGTCCACAGTTTAAGGAGTCC	GGTACGCCCACTGATGAGATT
OAS2	TTGAAGAGGAATACATGCGGAAG	GGGTCTGCATTACTGGCACTT
NF-kB	AGCGGGAACTGAGTGAGATGA	GCACCCAGGTTGTATCGGG
PKR	ATGCACGGAGTAGCCATTACG	TGACAATCCACCTTGTTTTCGT
RUNX1	CCATAGAGCCATCAAAATCAC	GCTTGGTCTGATCATCTAGTTTC
MMP1	CTTCTTCTTGTTGAGCTGGACTC	CTGTGGAGGTCACTGTAGACT
MMP2	ACCTGAACACTTTCTATGGCTG	CTTCCGCATGGTCTCGATG
MMP9	GGACCCGAAGCGGACATTG	CGTCGTCGAAATGGGCATCT
TLR2	CACCACTGCCCGTAGATGAAG	AGGGTACAGTCGTCGAACTCT
TLR3	AAAATCCTTGCGTTGCGAAGT	TGTTCAAGAGGAGGGCGAATAA
TLR7	CACCACCAATCTTACCCTTACC	CAGATGGTTCAGCCTACGGAA
TLR8	GTCCAAGGTGTTACAATGCTCC	AGCCAGAGGATGTATGTGGAT
TLR9	ACGGGAACTGCTACTACAAGA	CCCAGCTTGACAATGAGGTTAT
C3aR1	TCGATGCTGACACCAATTCAA	AGTCCCAATAGACAAGTGAGACC
C5a	GAACAAACCTACGTCATTTCAGC	CGGGGACAAATTAACATAGCCTG
C5aR1	TACCATTAGTGCCGACCGTTT	CCGGTACACGAAGGATGGAAT
TMEM119	TCTTCCGGCAGTACGTGATG	CGGCGCAGACTATGAACATGA
P2RY12	CCTGCCTTGATCCATTCATCTA	GTCCTTTCTTCTTGTTTGTCCC
TREM2	CTGGAACCGTCACCATCACTC	CGAAACTCGATGACTCCTCGG
CSF1R	CAGTTCAGAGTGATGTGTGGTC	CTTGTTGTTCACTAGGATGCCG
CVA2-VP1	TCAGTCCCATTCATGTCGCC	AATGCGTTGTTGGGGCATTG
Mouse β-actin	GTGCTATGTTGCTCTAGACTTCG	ATGCCACAGGATTCCATACC

Fig. S1

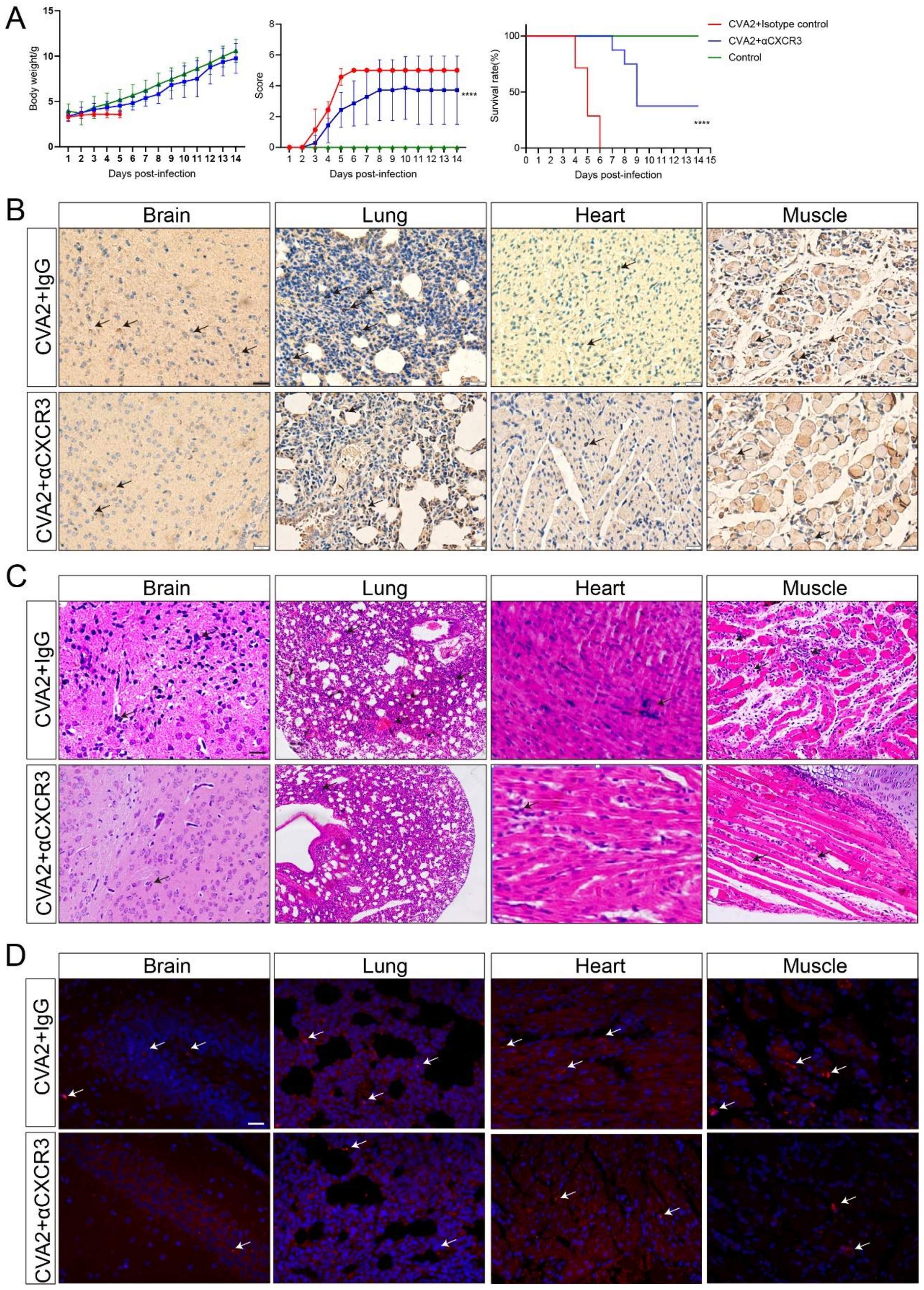

A
Body weight/g
Days post-infection
Score
****
Survival rate(%)
CVA2+Isotype control
CVA2+αCXCR3
Control
B
Brain
Lung
Heart
Muscle
CVA2+IgG
CVA2+αCXCR3
C
D

Fig. S2

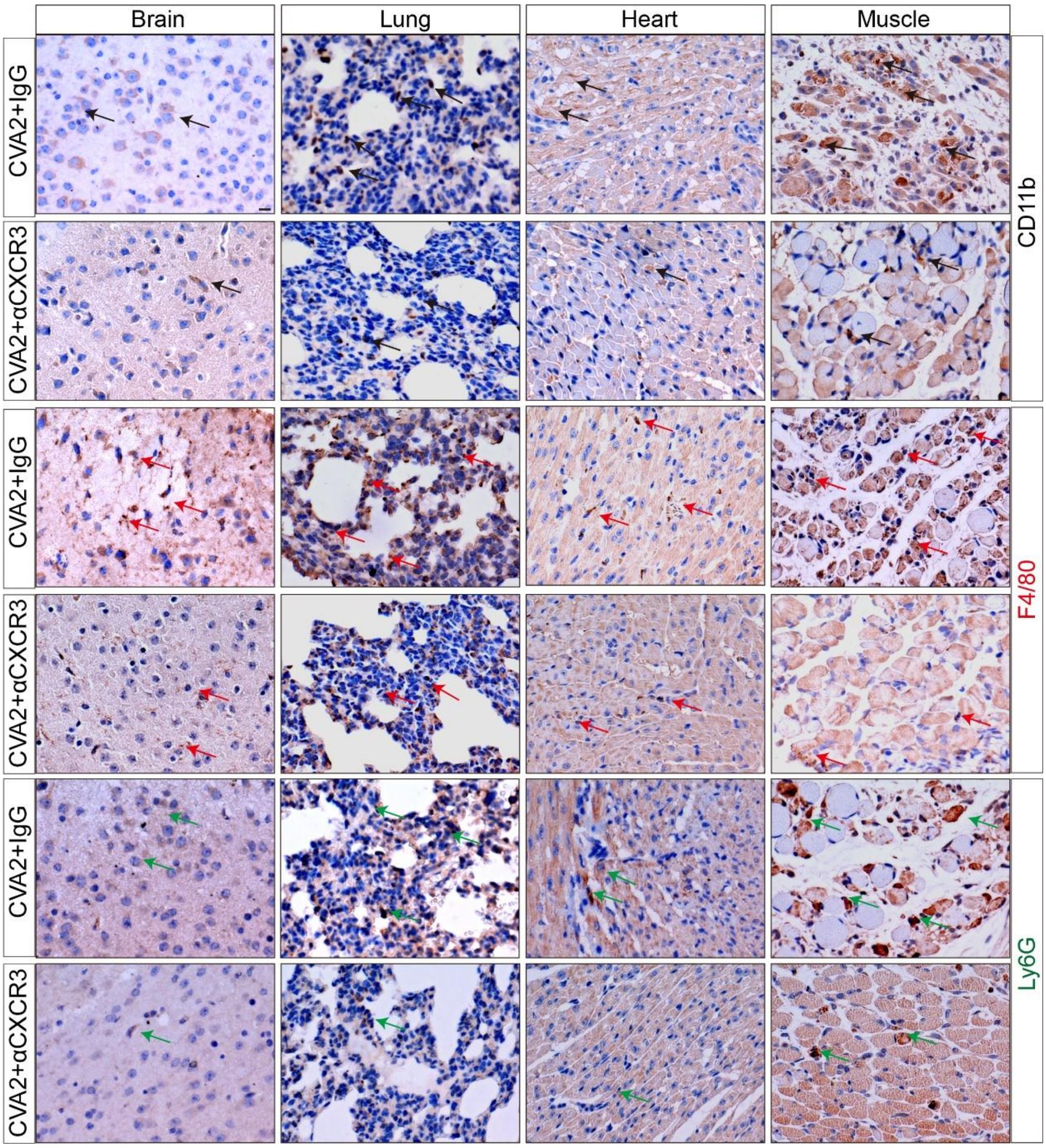

Figure legends

Fig. S1 The CXCL10/CXCR3 axis plays a crucial role in disease pathogenesis upon CVA2 infection. (A) Body weights, mean clinical scores and survival rates of IgG-treated groups after CVA2 infection, αCXCR3-treated groups after infection and control group, the IgG-treated mice did not survive longer than 5dpi and that the clinical scores on the days afterwards were carry-overs from 5pi. n = 8~10 per group,

**P < 0.01; ****P < 0.0001. (B) Immunohistochemical staining of viral antigen was conducted in the slices of brains, lungs, hearts, skeletal muscles. The black arrows indicate the locations of viral antigen, Bar = 50 um. (C) Histopathological changes of the organs and tissues (brains, lungs, hearts, skeletal muscles) in IgG Isotype and αCXCR3 groups (Bar = 1 mm). (D) Immunofluorescence staining of Cl-Caspase-3 (red) was conducted in the organs and tissues (Bar = 50 um).

Fig. S2 Blockade of CXCR3 causes alteration of inflammatory cells infiltration. IHC staining of CD11b, F4/80 and Ly6G in the organs and tissues from IgG isotype and αCXCR3-treated mice. Black arrows mark CD11b+ cells (mononuclear leucocytes); Red arrows mark F4/80+ cells (macrophages); Green arrows mark Ly6G+ cells (neutrophils) (Bar = 50 um).
